# Breastfeeding experiences and support for women who are overweight or obese: A mixed‐methods systematic review

**DOI:** 10.1111/mcn.12865

**Published:** 2019-08-06

**Authors:** Yan‐Shing Chang, Amaia Artazcoz Glaria, Philippa Davie, Sarah Beake, Debra Bick

**Affiliations:** ^1^ Florence Nightingale Faculty of Nursing, Midwifery and Palliative Care King's College London London UK; ^2^ Faculty of Life Sciences and Medicine King's College London London UK; ^3^ Institute of Psychiatry, Psychology and Neuroscience King's College London London UK; ^4^ Warwick Clinical Trials Unit University of Warwick Coventry UK

**Keywords:** body mass index, breastfeeding, breastfeeding experiences, breastfeeding support, obesity, overweight

## Abstract

Women who are overweight or obese have increased health risks during and beyond pregnancy, with consequences for their infants' shorter and longer term health. Exclusive breastfeeding to 6 months has many benefits for women and their infants. However, women who are overweight or obese have lower rates of breastfeeding intention, initiation, and duration compared with women with normal weight. This systematic review aimed to examine evidence of (a) breastfeeding barriers and support experienced and perceived by women who are overweight or obese, (b) support shown to be effective in increasing breastfeeding initiation and duration among these women, and (c) perceptions of health care professionals, peer supporters, partners, and family members regarding providing breastfeeding support to these women. Sixteen quantitative and qualitative papers were included and critically appraised. Thematic synthesis was undertaken to obtain findings. Maternal physical barriers such as larger breasts, difficulties of positioning to breastfeed, delayed onset of lactation, perceived insufficient supply of breast milk, and impact of caesarean birth were evident. Maternal psychological barriers including low confidence in ability to breastfeed, negative body image, embarrassment at breastfeeding in public, and experiencing stigma of obesity were also described. Support from health care professionals and family members influenced breastfeeding outcomes. Education for maternity care professionals is needed to enable them to provide tailored, evidence‐based support to women who are overweight or obese who want to breastfeed. Research on health care professionals, partners, and family members' experiences and views on supporting this group of women to breastfeed is needed to support development of appropriate interventions.

**Key messages**

- Physical and psychological barriers to initiate and continue breastfeeding were identified among women who are overweight or obese.
- Appropriate education and training are needed for maternity care professionals on how to improve and tailor support for women with a higher body mass index to breastfeed.
- Limited research was found of health care professionals, partners', and family members' perspectives on supporting women who are overweight or obese to breastfeed.
- Further robust research, with larger sample sizes, should be prioritised given the increasing burden globally of obesity among women of reproductive age.

## 1 INTRODUCTION

Prevalence rates of obesity and overweight among women of reproductive age are increasing. In the United Kingdom (UK), the proportions of women who were overweight or obese aged 16–24, 25–34, and 35–44 were 36%, 44%, and 57%, respectively, in 2016 (Health and Social Care Information Centre, 2017). In the United States, 55.8% of women aged between 20 and 39 years in 2009–2010 had a Body Mass Index (BMI) ≥25 kg/m^2^ (Flegal, Carroll, Kit, & Ogden, 2012). Overweight and obesity present health risks during and beyond pregnancy. Women with a pre‐pregnancy BMI ≥ 25 kg/m^2^ are significantly more likely to require induction of labour, intrapartum intervention, or caesarean section (elective and emergency; Marchi, Berg, Dencker, Olander, & Begley, 2015; Ovesen, Rasmussen, & Kesmodel, 2011; Poston et al., 2016; Sebire et al., 2001). For infants of women who are overweight or obese, there are higher risks of admission to neonatal units, macrosomia (birthweight >4,000 g) or birthweight above the 90th centile (large‐for‐gestational age; Marchi et al., 2015; Ovesen et al., 2011; Poston et al., 2016; Ruager‐Martin, Hyde, & Modi, 2010; Sebire et al., 2001), and higher BMI in childhood and young adulthood (Godfrey et al., 2017).

As breastfeeding significantly reduces the risk of children being overweight or obese, and developing associated diseases (Horta, Loret de Mola, & Victora, 2015; Martin, Gunnell, & Davey Smith, 2005), breastfeeding among women who are overweight or obese and their infants is particularly important. However, women with higher BMIs are less likely to initiate, continue, or exclusively breastfeed than women who have a “normal” BMI (BMI between 18.5 and 25.0 kg/m^2^; Amir & Donath, 2007; Mäkelä, Vaarno, Kaljonen, Niinikosk, & Lagström, 2014; Turcksin, Bel, Galjaard, & Devlieger, 2014; Wojcicki, 2011). Other potential benefits of breastfeeding for women include support for postnatal weight management (Baker et al., 2008; Vinter et al., 2014) and reduced risk of ovarian and breast cancer and Type 2 diabetes (Horta, Bahl, Martines, & Victora, 2007; Ip et al., 2007; Victora et al., 2016). Exclusively breastfed infants have reduced risk of contracting respiratory, gastrointestinal, and ear infections in infancy compared with infants not exposed to same levels of breastfeeding exclusivity or duration (Eidelman et al., 2012; Ip et al., 2007; Victora et al., 2016). Evidence for breastfeeding support available and experienced by women who are overweight or obese is limited.

This systematic review aimed to examine evidence of (a) breastfeeding barriers and support experienced and perceived by women who are overweight or obese, (b) support shown to be effective in increasing breastfeeding initiation and duration among women with higher BMIs, and (c) perceptions of health care professionals, peer supporters, partners, and family members regarding providing breastfeeding support to this group of women. The review was registered on PROSPERO: CRD42016039916.

## 2 METHODS

An “integrated methodology” was adopted (Joanna Briggs Institute, 2014; Sandelowski, Voils, & Barroso, 2006) in which findings of qualitative and quantitative studies can confirm or refute each other, with data assimilated into one single synthesis. The review was designed to answer the following questions:

- What are perceptions and experiences of breastfeeding barriers among women who are overweight or obese?
- What are these women's experiences of support for breastfeeding offered by health care professionals, peer supporters, and family members during and after pregnancy, including type and content of support?
- What types and content of support offered by health care professionals, peer supporters, and family members during and after pregnancy could increase breastfeeding initiation and continuation among women who are overweight or obese?
- What are health care professionals', peer supporters', and family members' perceptions of providing breastfeeding support and how do they perceive their role in this?

### 2.1 Eligibility criteria

The PICOS (Population/Participants, Interventions/Phenomena of interest, Comparison/Context, Outcomes, and Study types) framework adapted from Joanna Briggs Institute (2014) and advocated for in the Preferred Reporting Items for Systematic Reviews and Meta‐Analyses (Moher et al., 2009) was used to develop the eligibility criteria to address the review questions as follows:

#### 2.1.1 Population/participants

Pregnant and postnatal women classed as overweight (BMI ≥ 25 kg/m^2^) or obese (BMI ≥ 30 kg/m^2^) as defined by study authors and those who offered breastfeeding support including partners, family, health care professionals, breastfeeding peer supporters, and lactation specialists were included.

#### 2.1.2 Interventions/phenomena of interest

Studies were included if they explored experiences and perceptions of breastfeeding and breastfeeding support, evaluations of breastfeeding interventions/support, and studies which considered experiences, perceptions, and information/training needs of those who offered support. Studies targeted at *all* women, irrespective of BMI, were excluded, as were studies where the primary aim was to establish breastfeeding initiation and duration among women who were overweight or obese which did not present (a) research data on barriers or facilitators to these or (b) evaluate the intervention/support provided.

#### 2.1.3 Comparison/context

For experimental/quasi‐experimental studies, comparisons could include usual care or a control group designed as a comparison to the described intervention. For nonexperimental studies, comparisons could include women who were not overweight or obese. Studies conducted in acute and/or primary care settings, communities, or participants' homes were included.

#### 2.1.4 Outcomes

Outcomes for intervention studies (as defined by study authors) included the following:

- rates of breastfeeding initiation;
- duration of exclusive breastfeeding; and
- duration of any breastfeeding.

Other outcomes, including for nonintervention studies, included the following:

- women's experiences and perceptions of support for breastfeeding provided by health care professionals, peers, and family members;
- maternal and infant physical and psychological factors that affected women's breastfeeding outcomes;
- experiences and views of those who supported women to breastfeed;
- women's confidence, knowledge, attitudes, and skills;
- supporters' (including professionals, peers, and family members) knowledge, attitudes, skills, and information/training needs;
- breastfeeding problems; and
- barriers to provision of interventions/support.

#### 2.1.5 Study types

Experimental (e.g., randomised controlled trials and cluster‐randomised trials) and quasi‐experimental studies were considered. For nonintervention studies, qualitative, quantitative, and mixed‐methods research papers presenting primary data and/or secondary data analysis using quantitative datasets were included. Reviews, dissertations, opinion pieces, guidelines, and policy papers were excluded. Studies published in English from January 1992 (following the launch of UNICEF's Baby Friendly Initiative) to October 2018 were included. Intervention studies published before 2014 were not considered as an earlier review of interventions to increase breastfeeding among women with higher BMIs only included studies up to 2013 (Babendure, Reifsnider, Mendias, Moramarco, & Davila, 2015).

### 2.2 Search strategy

**Figure 1:**
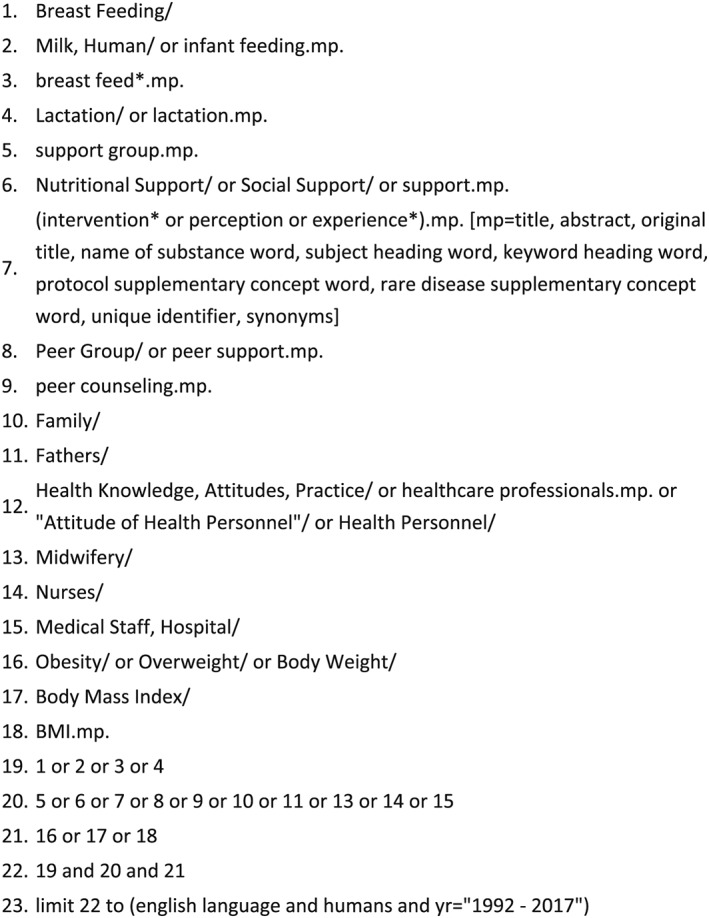
Search strategy example (Medline)

A search of Medline, Embase, Maternity and Infant Care, CINAHL, SCOPUS, PsycInfo, Web of Science, and Cochrane Library was conducted using search terms and Medical Sub‐Headings (MeSH) terms. Searches were undertaken to identify unpublished studies and reports published in grey literature sources including OpenGrey and websites of organisations which support breastfeeding and/or weight management such as WHO, UNICEF, La Leche League International, and commercial weight management programmes. Reference lists of selected papers and identified reviews were searched for additional papers. Initial key words and indexed terms included obesity, overweight, breastfeeding, lactation, and support. MeSH terms were identified through reading published studies and use of the MeSH terms lookup tool in the Cochrane Library. Figure 1 shows an example of a full search strategy for Medline.

### 2.3 Study selection

All identified papers were initially screened for relevance based on title and date published and then further assessed by reading the abstract. Full texts were then retrieved and assessed against eligibility criteria. Full texts were assessed by AGG, Y‐SC and SB and verified by DB. Any disagreements were resolved through discussion.

### 2.4 Quality assessment and data extraction

The Critical Appraisal Skills Programme's (CASP) critical appraisal checklists (CASP, 2017) were adapted for quality assessment of qualitative research, case‐control, and cohort studies. Each CASP question applied to the paper was answered “yes” or “no” or “cannot tell.” A “yes” response was allocated one point, with no points for “no” or “cannot tell.” Points were then added up to a total score for each paper. A maximum score of 10 could be allocated for the CASP checklist of qualitative research, 13 for case‐control studies, and 14 for cohort studies. In the absence of a suitable CASP checklist for cross‐sectional studies, a checklist for questionnaires and surveys (Greenhalgh, Robert, Bate, Macfarlane, & Kyriakidou, 2005) was used, with a maximum score of 13. Quality assessment was independently conducted by AGG, PD, SB and verified by Y‐SC and DB. Any disagreements were resolved through discussion. Studies which scored less than 8 on relevant appraisal tools were excluded.

Data were extracted from included studies by AGG, PD, SB and Y&SC. DB and Y&SC verified the extracted data and corrected where necessary. Two data extraction forms, which were adapted from the authors' previous published systematic reviews, were used (Beake et al., 2017; Beake et al., 2018). One form is for quantitative studies and the other is for qualitative studies. Data extraction for quantitative studies included aim/objectives, study design, setting, participants, inclusion/exclusion criteria, outcome measures, intervention, results, and additional analysis, for example, subgroups. Data extraction for qualitative studies included aim/phenomena of interest, methodology, setting, participants, sampling methods, data collection, data analysis, and results. The key characteristics (study aim, study methods, sample, and key findings) for each included paper are presented in Table 1.

**Table 1:** Characteristics of the included studies

Authors, year (country)	Aim of quantitative study	Study design and critical appraisal score	Study sample	Key results
Hauff & Demerath, 2012 (USA)	To test differences in breastfeeding duration by pre‐pregnant maternal weight status, and identify whether body image concerns mediate any differences.	A prospective longitudinal cohort study. CASP cohort study checklist: Yes 12/14 No 2/14	257 primiparous women intending to breastfeed. Maternal BMI based on participants' self‐reported pre‐pregnancy weight and height.	Women who were overweight or obese were significantly more likely to report “not feeling comfortable/confident in their bodies” at 4‐month postpartum compared with women with normal BMIs. Reduced breastfeeding duration by women who were overweight or obese appeared to be mediated by this postpartum lack of comfort/confidence.
Hauff et al., 2014 (USA)	To determine associations of maternal obesity and psychological factors with breastfeeding outcomes.	A prospective longitudinal cohort study. CASP cohort study checklist: Yes 12/14 No 2/14	2,824 women taking part in the nation‐wide infant feeding practices study II (IFPS II). Maternal BMI based on participants' self‐reported pre‐pregnancy weight and height.	Maternal pre‐pregnancy BMI was significantly associated with social knowledge of breastfeeding, social influence towards breastfeeding and maternal confidence in reaching her breastfeeding duration goal. Maternal attitudes and behavioural beliefs towards breastfeeding were not significantly associated with maternal pre‐pregnancy BMI.
Jarlenski et al., 2014 (USA)	To compare (a) barriers to breastfeeding among women who were obese versus women who were not obese(b) the association between breastfeeding support from a physician or other health professional and women's breastfeeding knowledge, initiation and duration, between women who were and were not obese.	A prospective longitudinal cohort study. CASP cohort study checklist: Yes 12/14 No 2/14	2,997 women taking part in the nation‐wide infant feeding practices study II (IFPS II). Maternal BMI based on participants' self‐reported pre‐pregnancy weight and height.	Most common reason for not initiating breastfeeding in both groups was “formula is the same or better than breastmilk”. Reasons for not initiating breastfeeding were comparable between women who were or were not obese, except significantly more women who were not obese reported “wanting body back to self” as a reason. Most common reason for not continuing breastfeeding for 6‐months was “breast milk alone did not satisfy the baby” with significantly more women who were obese reporting this. “Did not have enough milk” was the second most common reason, with significantly more women were obese reporting this. Significantly more women who were obese reported “baby had trouble sucking or latching on”. Support: In the full sample support for exclusive breastfeeding from physicians was significantly associated with an increased likelihood that women would initiate breastfeeding and would continue breastfeeding for 6 months. Support from a healthcare practitioner significantly increased initiation and the likelihood to continue breastfeeding to 6 months.
Kair & Colaizy, 2016a. (USA)	To identify barriers to breastfeeding continuation among women who were overweight and obese.	A retrospective cohort study. CASP cohort study checklist: Yes 12/14 No 2/14	6,467 women who initiated breastfeeding, but discontinued before survey completion at 4 months postpartum (part of 19,145 women recruited through the Pregnancy Risk Assessment Monitoring System (PRAMS). Maternal BMI was based on participants' self‐reported pre‐pregnancy weight and height.	The most common reasons in all BMI groups for discontinuing breastfeeding were “not producing enough milk,” “breast milk alone did not satisfy my baby,” and “baby has difficulty nursing.” Women who were overweight compared to women with normal BMIs had significantly higher chances of discontinuing breastfeeding due to ‘breastmilk alone did not satisfy [their] infant'. This remained significant in adjusted models controlling for covariates. Women who were obese compared to women with normal BMIs had significantly greater odds of discontinuing breastfeeding due to “breast milk along did not satisfy [their] infant”, “difficulty nursing' their infants, and because their babies were “jaundice”. Women who were obese compared to women with normal BMI, had significantly lower odds of discontinuing breastfeeding due to “sore, cracked or bleeding nipples”, or “it was the right time”. These remained significant in adjusted models for covariates.
Kair & Colaizy, 2016b. (USA)	To examine the extent to which a woman's pre‐pregnancy BMI category is associated with her exposure to pro‐breastfeeding hospital practices.	A cross‐sectional study. Greenhalgh (2015) checklist for questionnaire surveys: Positive assessment 10/13 Cannot tell 2/13 Negative assessment 1/13	19,145 women recruited through the pregnancy risk assessment monitoring system (PRAMS) national data surveillance project carried out by the United States Centers for Disease Control and Prevention. Maternal BMI was based on participants' self‐reported pre‐pregnancy weight and height.	Women who were obese, compared to women with normal BMIs, had significantly lower chances of being provided breastfeeding information, rooming in with their infants, breastfeeding in hospital, breastfeeding within 1 hr delivery, having staff help them breastfeed, feeding exclusive breast milk in hospital. Having advice to breastfeed on, or being given a telephone number for BF support, and significantly higher chances of their infants using a pacifier. These remained significant in adjusted models (except for rooming in).
Lau et al., 2017 (Singapore)	To examine the relationships among maternal characteristics, health‐related quality of life and breastfeeding attitude among women with normal and overweight/obese BMIs	An exploratory cross‐sectional study. Greenhalgh (2015) checklist for questionnaire surveys: Positive assessment 8/13 Cannot tell 3/13 Negative assessment 2/13	708 pregnant women (78.8% response rate). Maternal BMI was based on participants' self‐reported pre‐pregnancy weight and height.	Better health‐related quality of life, higher monthly household income, planned pregnancy and previous exclusive breastfeeding experiences were significantly associated with positive breastfeeding attitude among women with normal and overweight/obese BMIs. Pregnant women with normal BMIs who were older with higher educational level were significantly more likely to have positive breast‐feeding attitude. Pregnant Chinese women who were overweight/obese with confinement nanny plan were significantly less likely to have positive breastfeeding attitude.
Mok et al., 2008 (France)	To compare (a) breastfeeding practices (b) perceptions of breastfeeding (c) infant weight change, between women with obese and normal BMIs, from birth to 3‐months postpartum.	A prospective case‐control matched‐pairs study. CASP case‐control study checklist: Yes 11/13 No 2/13	222 women who gave birth in Poitiers (111 women who were obese matched with 111 women with normal BMIs).	The choice of how to initially feed her infant was significantly influenced by the feeding practices of close family members and opinions of the father among all women. Significantly more women who were obese compared to women with normal BMIs reported difficulties with breastfeeding in hospital, at 1‐month postpartum and at 3‐month postpartum. Significantly more women with normal BMIs perceived having an adequate milk supply at 1‐month postpartum and at 3‐month postpartum. Inadequate milk supply was the main reason for breastfeeding cessation among breastfeeding women who were obese. A greater proportion of women who were obese than women with normal BMIs reported feeling uncomfortable breastfeeding in the presence of others at 1 month and a significantly greater proportion at 3 months. Women who were obese compared to women with normal BMIs were less often followed by HCP or organisations for breastfeeding support on maternity wards, at 1‐month and 3‐month postpartum.
Nommsen‐Rivers et al., 2010 (USA)	To examined variables associated with delayed onset of lactogenesis among first‐time mothers who delivered at term and initiated breastfeeding	A prospective longitudinal cohort study. CASP cohort study checklist: Yes 12/14 No 2/14	431 primiparous women. Maternal BMI was based on participants' weight and height measured by interviewer at the day 7 postpartum visit.	Maternal postpartum BMI was significantly associated with a delayed onset of lactogenesis II. Delayed lactogenesis was lowest in women with normal BMIs, then women who were overweight, and highest among women who were obese. In adjusted multivariate logistic regression models, maternal overweight and obesity were independently, significantly associated to delayed onset of lactogenesis II when compared to women with normal BMIs.
O'Sullivan et al., 2015 (USA)	To determine whether the negative association between obesity and any or exclusivebreastfeeding at 1 and 2 months postpartum is mediated through breastfeeding problems occurring in the first 2 week postpartum and if this association differs by parity.	A prospective longitudinal cohort study. CASP cohort study checklist: Yes 10/14 Cannot tell 2/14 No 2/14	1,731 women taking part in the nation‐wide infant feeding practices study II (IFPS II) (1151 women with normal BMIs, 580 women who were obese)	No significant effect of obesity was found on any breastfeeding at 1 or 2 months. At 1 month postpartum, for both primiparous and multiparous women, there was a significant direct effect of obesity on exclusive breastfeeding and a significant indirect effect of obesity through early breastfeeding problems related to ‘insufficient Milk'. At 2 months postpartum both the direct effect of obesity and indirect effect of ‘insufficient Milk' were significant in primiparous women. Only the indirect effect remained significant in multiparous women.
Swanson et al., 2017 (UK)	To compare between women who were obese and women with healthy weight, the relationships between women's body image and breastfeeding outcomes, and how postnatal psychological distress was related to body image and breastfeeding maintenance.	A prospective cohort study. CASP cohort study checklist: Yes 11/14 Cannot tell 3/14	140 postnatal women. 70 healthy weight (BMI at any stage of pregnancy of 18.5 < 25 kg/m^2^) and 70 women who were obese (BMI at any stage of pregnancy >30 kg/m^2^). Women's BMI were taken from case notes.	Women with healthy weight were more likely to exclusively breastfeed in hospital, and maintain breastfeeding at 6–8 weeks than women who were obese. Body image was lower overall in women who were obese. All body image components, except appearance orientation, were correlated with breastfeeding maintenance and weight status. Higher satisfaction and appearance evaluation were positively related to breastfeeding and negatively related to weight status.
Claesson et al., 2018 (Sweden)	To identify and describe breastfeeding experiences of women who were obese	Semistructured face‐to‐face interviews. CASP qualitative study checklist: Yes 9/10 No 1/10	11 women who were obese (2–18 months postpartum). Self‐reported pre‐pregnancy BMI ≥ 30 kg/m^2^.	3 main themes: (1) breastfeeding – a part of motherhood (2) the challenges of breastfeeding (3) support for breastfeeding. Challenges included technical difficulties and exposure of the body in public. Support included the importance of being an individual behind the obesity and to obtain enough professional support.
Garner et al., 2014 (USA)	To describe the experiences of healthcare professionals providing breastfeeding support for women who were obese in the pre‐, peri‐ and post‐natal periods.	In‐depth, face‐to‐face, semi‐structured interviews. CASP: Qualitative study checklist: Yes 10/10	34 healthcare professionals (HCP): 4 obstetricians, 4 paediatricians, 3 family medicine physicians, 5 nurse midwives, 2 nurse practitioners, 8 registered nurses, 8 lactation consultants.	4 main themes: (1) Identification of obesity (2) HCPs perception of challenges for women who were obese in breastfeeding (3) Challenges for HCPs (4) Improving breastfeeding care for women who were obese. HCPs perceived breastfeeding care for women who were obese to require more time, more physical effort and posed as more of a challenge than women who were not obese. Breastfeeding support was not a priority due to extra time needed to address comorbidities. Lots of HCPs were unsure of how to improve care and reported they needed more education.
Garner et al., 2017 (USA)	To understand breastfeeding experiences and perceptions among women who were obeselongitudinally, with a comparison group of women with normal BMIs.	Semistructured interviews. Interviews during pregnancy and postpartum 7–10 days, 6 weeks, 3 months and an optional 6‐month phone call. CASP qualitative study checklist: Yes 9/10 No 1/10	13 women who were obese (BMI ≥ 30 kg/m^2^ self‐reported at third trimester), 9 women with normal BMIs (BMI 18.5–24.9 kg/m^2^, self‐reported at third trimester). All participants intended to breastfeed.	5 main themes; (1) breastfeeding plans and confidence (2) breastfeeding affected by health issues (3) positioning and latching (4) nursing bras (5) social support. Compared to women with normal BMIs, women who were obese expressed less breastfeeding confidence, experienced more latching and positioning challenges, more health issues affecting breastfeeding, more difficulties finding nursing bras, and required more tangible support.
Keely et al., 2015 (UK)	To explore factors influencing breastfeeding practices among women who were obese.	Semistructured, in‐depth face to face interviews at 6–10 weeks postpartum. CASP qualitative study checklist: Yes 9/10 No 1/10	28 women who were obese (BMI >30 kg/m^2^), who initiated breastfeeding, but had stopped (at all or exclusively) breastfeeding at 6–10 weeks postnatal, despite an original intention to exclusively breastfeed for at least 16 weeks.	3 main themes: (1) impact of birth complications (e.g. caesarean section) on breastfeeding (2) lack of privacy as a barrier to breastfeed (3) breastfeeding support including subthemes of physical difficulties, early introduction of formula, role of partners, breastfeeding clinics, and other sources of support.
Massov, 2015 (New Zealand)	To describe breastfeeding experiences and perceptions, and influences on infant feeding decisions among women who were obese.	Semistructured, in‐depth face to face interviews during postpartum period. CASP: Qualitative study checklist: Yes 9/10 No 1/10	6 women who were obese (BMI >30 kg/m^2^ at antenatal booking visit), who had initiated breastfeeding but ceased breastfeeding fully or exclusively 4–6 weeks postpartum.	7 themes: (1) breastfeeding as difficult (2) insufficient breastmilk supply (3) physical difficulties ‐ latching and mechanical factors (4) unrealistic expectations (5) pressure to breastfeed (6) professional help as distressing (7) being philosophical about their decision. Women reported they were happy with persevering as long as they did to give their babies the optimum nutrition in the beginning. The decision to supplement feed was rationalised as a decision for their infants' overall health and well‐being.
McKenzie et al., 2018 (USA)	To describe (a) U.S. women's experiences with breastfeeding in public (b) experiences of women who were obese compared with those of women with normal BMIs.	Semistructured interviews. Interviews during pregnancy and postpartum 7–10 days, 6 weeks, 3‐months and an optional 6‐month phone call. CASP qualitative study checklist: Yes 10/10	13 women who were obese (BMI ≥ 30 kg/m^2^ self‐reported at third trimester), 9 women with normal BMIs (BMI 18.5–24.9 kg/m^2^, self‐reported at third trimester). All participants intended to breastfeed.	4 main themes (1) “public' can be anywhere (2) social awkwardness of breastfeeding around others (3) physical awkwardness of breastfeeding around others (4) coping strategies. Women who were obese experienced challenges to a greater degree than women with normal BMIs.

### 2.5 Data synthesis

In line with an “integrated methodology,” quantitative and qualitative data were assimilated into a single synthesis. Using this approach, studies are grouped for synthesis using findings which answer the same review questions, rather than by study methods, enabling integration of findings (Dixon‐Woods, Agarwal, Jones, Young, & Sutton, 2005). Findings from quantitative data were extracted narratively, “converted” into themes and integrated with qualitative data. Thematic synthesis steps adapted from Lucas, Baird, Arai, Law, and Roberts (2007) and Smith, Begley, Clarke, and Devane (2012) were adhered to, namely,

1. data were extracted from findings of included studies;
2. extracted data were grouped for each review question and emergent themes identified;
3. a list of themes was presented for each question; and
4. a synthesis of findings was produced.

Due to differences in quantitative study designs and outcomes, meta‐analysis could not be performed.

## 3 FINDINGS

**Figure 2:**
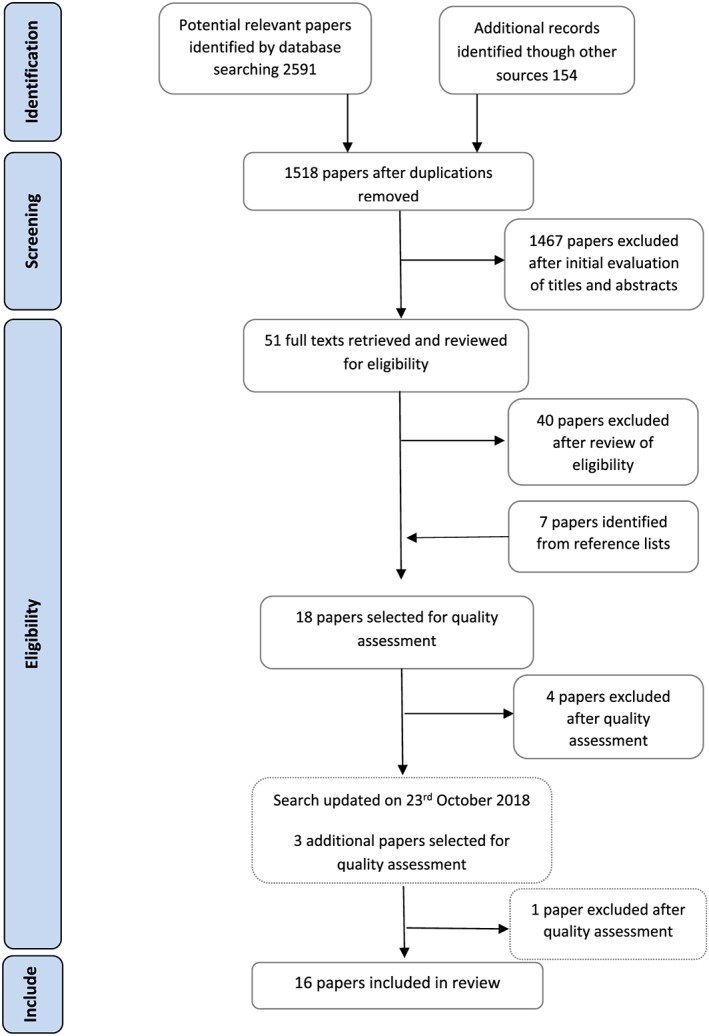
Flow chart of stages of searching

Following the initial systematic search on September 2, 2017, 2,591 publications were identified (see Figure 2). After removing duplicates, 1,518 remained. Titles were screened for relevance after which 220 abstracts were obtained for further screening by AGG and Y‐SC. Following title and abstract screening, 51 full texts were retrieved and read by AGG and Y‐SC. Forty papers were excluded which did not address the review questions. Reference lists of selected papers and relevant reviews were searched, and seven further papers were identified. Searches were updated on October 23, 2018, and three additional articles were selected for quality assessment. Quality assessment was conducted for 21 papers using the appropriate critical appraisal checklist. Following quality assessment, five papers were excluded (Katz, Nilsson, & Rasmussen, 2009; Lewkowitz et al., 2018; Newby & Davies, 2016; Rasmussen, Lee, Ledkovsky, & Kjolhede, 2006; Zanardo et al., 2014) due to poor quality of data presented. Quality assessment scores of the final included papers are included in Table 1.

Sixteen papers were included: six qualitative studies, six prospective cohort studies, one retrospective cohort study, one case‐control study, and two cross‐sectional studies. Two papers (Garner, McKenzie, Devine, Thornburg, & Rasmussen, 2017; McKenzie, Ramussen, & Garner, 2018) were from the same study. All papers were from high‐income countries: 10 from the United States, two from UK, and with single papers from France, New Zealand, Singapore, and Sweden. One paper focused on health care professionals, all others explored women's experiences of breastfeeding, perceptions of support offered, perceptions of body image, breastfeeding practices, and views of barriers to breastfeeding.

Only two papers (Garner, Ratcliff, Devine, Thornburg, & Rasmussen, 2014; McKenzie et al., 2018) achieved a full CASP score of 10. The other studies had methodological limitations, including exposure variables which may not have been accurately measured to minimise bias (Hauff & Demerath, 2012; Hauff, Leonard, & Rasmussen, 2014; Jarlenski et al., 2014; Kair & Colaizy, 2016a; Nommsen‐Rivers, Chantry, Peerson, Cohen, & Dewey, 2010; O'Sullivan, Perrine, & Rasmussen, 2015). Two qualitative studies (Garner et al., 2017; Keely, Lawton, Swanson, & Denison, 2015) were allocated lower scores as the relationship between researcher and study participants was not explained.

### 3.1 What are perceptions and experiences of breastfeeding barriers among women who are overweight or obese?

Included studies reported many physical and psychological barriers to breastfeeding among women with higher BMIs, which are considered in the following sections.

#### 3.1.1 Positioning and attaching to the breast

Quantitative and qualitative studies reported physical barriers including larger breasts, bigger areolas, and additional body tissue made infant handling and breastfeeding positions such as cradle or cross cradle more difficult (Claesson, Larsson, Steen, & Alehagen, 2018; Garner et al., 2017; Jarlenski et al., 2014; Massov, 2015). Jarlenski et al. (2014) found that significantly more women with obesity (26.5%) than without obesity (21.0%; *p* < .05) reported “baby had trouble sucking or latching on” as a reason for not breastfeeding to 6 months (Jarlenski et al., 2014).

Garner et al.'s (2017) qualitative study further found that women with obesity reported breastfeeding took more time, including preparing to feed, and required more physical “props,” such as pillows, limiting places where they felt able to breastfeed outside of the home. Finding nursing bras to fit was also identified as a problem for them. Additionally, Massov (2015) reported women's concerns that as their breasts were heavy, they were worried they would suffocate their infant by “squishing” them.

#### 3.1.2 Breast problems

In a matched case‐control study from France (Mok et al., 2008), a significantly higher proportion of women with obesity (56.7%) reported physical difficulties with breastfeeding (i.e., cracked nipples and fatigue or difficulty initiating a breastfeed) in hospital, compared with normal BMI women (13.3%; *p* < .05). Kair and Colaizy (2016a) reported findings from a large retrospective cohort study of women's reasons for stopping breastfeeding in the United States. Compared with women with normal weight who breastfed, women with obesity had significantly *higher* odds of reporting sore, cracked, or bleeding nipples (OR = 0.70, 95% CI [0.54, 0.91], *p* = .008) and *lower* odds of reporting that they stopped breastfeeding when they felt it was the best time for them to stop (OR = 0.69, 95% CI [0.49, 0.96], *p* = .028), which suggested their desire to breastfeed for a longer duration.

#### 3.1.3 *Delayed onset of lactation* and *perceived insufficient breast milk*

Being overweight or obese was an independent risk factor for delayed onset of lactation (Nommsen‐Rivers et al., 2010), with “delayed” defined as breasts being “noticeably fuller” after 72‐hr postpartum. Women's perceptions of insufficient breastmilk supply have been reported as a key factor for stopping breastfeeding (Jarlenski et al., 2014; Kair & Colaizy, 2016a; Massov, 2015; Mok et al., 2008; O'Sullivan et al., 2015). For example, Jarlenski et al. (2014) reported perceptions of low breastmilk supply as a reason for early cessation among women with and without obesity, with more women with obesity (55.5%) reporting this than women without obesity (48.3%; *p* < .05). “Did not have enough milk” was the second most common reason provided in both groups, but significantly more women with obesity (51.3%) reported this than women without obesity (45.0%; *p* < .05). The women in Massov's (2015) study described perceived insufficient breast milk supply as a reason for switching to formula feeding. Claesson et al.'s (2018) qualitative study described how women thought that having larger breasts might impair milk production. O'Sullivan et al. (2015) found that obesity negatively affected exclusive breastfeeding, and the association was significantly mediated by the perception of “insufficient milk” supply.

#### 3.1.4 Impact of caesarean birth

Having a caesarean birth was identified as a specific barrier for women with obesity to breastfeed (Garner et al., 2014; Garner et al., 2017; Keely et al., 2015). Women who had a caesarean birth considered that anaesthetic drugs made it harder for them to think and react properly in the post‐operative period and that caesarean birth delayed skin to skin care, presenting a barrier to breastfeeding initiation (Keely et al., 2015). Women's limited mobility following a caesarean birth was reported as a perceived barrier to breastfeeding by clinicians interviewed by Garner et al. (2014), and experiences of poor post‐caesarean health and recovery (such as developing severe infections) were described as barriers by women with obesity (Garner et al., 2017).

#### 3.1.5 Attitudes and low confidence in ability to breastfeed

Hauff et al. (2014) showed maternal BMI was significantly associated with maternal confidence in achieving breastfeeding duration goals (*p* < .0001). A higher proportion of women with obesity (10.3%) rated they were “not confident” in their ability to breastfeed for as long as planned, compared with women with overweight BMIs (8.8%) or normal BMIs (5.4%). Women who were not confident they would achieve their breastfeeding goals were significantly more likely to stop breastfeeding earlier than women who were confident (HR: 2.50 95% CI [2.07, 3.02]). However, maternal attitudes and beliefs towards breastfeeding were not significantly different among women with normal, overweight, or obese BMIs (*p* = .40). Similarly, Lau et al. (2017) found that attitudes to breastfeeding were comparable among women with normal and overweight/obese BMIs (*p* = .851) in their study.

#### 3.1.6 Body image

Two studies (Hauff & Demerath, 2012; Swanson, Keely, & Denison, 2017) investigated the relationships between women's perceptions of body image and breastfeeding. Hauff and Demerath (2012) found that women who were overweight or obese were significantly more likely to report not feeling body confident (50%, *n* = 38) at 4 months postnatally, compared with 28.5% (*n* = 45) of women with normal BMIs (*p* = .001), and feeling body confident was significantly associated with both exclusive (*p* < .001) and any breastfeeding (*p* < .001) at 4‐month postpartum. Women's lack of body comfort/confidence was found to significantly mediate the relationship between maternal obesity and reduced duration of any breastfeeding. Swanson et al. (2017) reported that women's perceptions of their body image was relatively low for all women in the postpartum period, but women with obesity were found to have significantly lower body satisfaction at 6‐8 weeks postpartum than healthy weight comparisons (*p* = .03). Body satisfaction was found to significantly mediate the relationship (*p* = .002) between weight status and any breastfeeding at 6–8 weeks.

#### 3.1.7 Breastfeeding in public

Embarrassment about breastfeeding in public was a key issue affecting breastfeeding behaviour (Claesson et al., 2018; Keely et al., 2015; Massov, 2015; McKenzie et al., 2018; Mok et al., 2008). Mok et al. (2008) reported that at 1‐month postpartum, a higher proportion of women with obesity (47%, *n* = 20) reported feeling uncomfortable when breastfeeding in the presence of others than women with normal weight (26%, *n* = 13), but this was not statistically significant. However, at 3‐month postpartum, significantly more women with obesity (42%) continued to report this, compared with women with normal body weight (13%; *p* < .01).

A woman in the study by Massov (2015) directly attributed lack of breastfeeding success due to her inability to be discreet when breastfeeding in public as her breasts were so large: “Yes, me personally, I'm just too self‐conscious to, because they're so big, to actually get them out in public” (Massov, 2015, p. 26). Keely et al. (2015) reported feeding in public was a source of anxiety for women, and women who decided to bottle feed felt comforted at not having to reveal their bodies. The open postnatal ward environment with a constant stream of visitors offered little privacy. The women who had a caesarean birth and required longer in‐patient stay found breastfeeding distressing due to a lack of privacy when sharing a room with other women, their partners, and visitors. Problems with privacy persisted at home, due to well‐intentioned frequent visits from family members and friends, as women faced the same potential for embarrassment at having to expose their bodies in front of them (Keely et al., 2015). Nevertheless, for some women, the awkwardness of breastfeeding around others could reduce over time: “now that [infant] can justlatch on and eat, I don't feel nearly as self‐conscious” (McKenzie et al., 2018, p. 764).

#### 3.1.8 Stigma associated with obesity

Hauff and Demerath (2012) reported stigma of obesity as a direct cause of poorer breastfeeding behaviours, including reduced duration. Kair and Colaizy (2016b) suggested that women who were overweight or obese were less likely to receive pro‐breastfeeding support in hospital than women with normal weight as a consequence of obesity stigma among hospital staff.

### 3.2 What are the experiences of support for breastfeeding offered by health care professionals, peer supporters, and family members during and after pregnancy among women who are overweight or obese, including type and content of support?

Studies of support for breastfeeding described women's positive and negative experiences of support offered and received.

#### 3.2.1 Social knowledge and support

Hauff et al. (2014) found a significant association between maternal BMI status and social knowledge of breastfeeding, that is, how many of women's friends or relatives had previous breastfeeding experience. Women with obesity were less likely to know any women with previous breastfeeding experience (18.7%) or knew only one to two women with previous experience (23.6%), when compared with women with overweight BMIs (13.7% and 21.7%, respectively) or normal BMIs (11.4% and 20.9%, respectively). Women in Keely et al.'s (2015, p. 536) study commented that their partners did not understand the frequency with which infants required feeding and expressed concerns that infants were not receiving adequate breast milk “I don't think [my husband] quite understood about the breastfeeding – that it is normal every half an hour and it is normal for [the baby] to cry.”

#### 3.2.2 Health care professionals' attitudes and practices

Kair and Colaizy (2016b) found the amount of breastfeeding support offered by health professionals differed according to women's BMI category. Compared with women with normal BMIs, in unadjusted models, women with obesity had lower odds of a staff member offering them information about breastfeeding (OR = 0.71. 95% CI [0.57, 0.89], *p* = .002), a staff member helping them to breastfeed (OR = 0.69, 95% CI [0.61, 0.78], *p* < .001), breastfeeding within an hour of the birth (OR = 0.55, 95% CI [0.49, 0.62], *p* < .001), being offered a telephone number for breastfeeding help (OR = 0.65, 95% CI [0.57, 0.74], *p* < .001), rooming in with their baby (OR = 0.84. 95% CI [0.73, 0.97], *p* = .02), or being informed to breastfeed on demand (OR = 0.66, 95% CI [0.58, 0.75], *p* < .001). All associations remained significant after adjusting for multiple covariates, except the association for “rooming in”. Jarlenski et al. (2014) found no differences between women with and without obesity in reporting that their physicians (*p* = .93) and other health care professionals (*p* = .51) supported/favoured exclusive breastfeeding.

Women found it helpful to receive regular home contacts with health care professionals. Keely et al. (2015) reported feedback from one woman who felt that the regular home contacts she received from a clinical assistant were vital to establishing a good breastfeeding routine. Women's self‐confidence increased when health professionals paid attention to them and that they were treated as an individual rather than an individual with obesity “they looked into my eyes and saw me as I was. Nobody focused on what I looked like …” (Claesson et al., 2018: 7). However, some women received judgemental and disempowering support from health care professionals. Massov (2015, p. 27) reported one woman with obesity who experienced “rough and aggressive” treatment: “I remember the midwife coming in and almost angry that I was upset because I was having trouble doing it ….” Another woman reported her experience of midwifery support as disempowering, as rather than showing her how to attach her baby to the breast, she felt midwives “were taking over”. Lacking support from health professionals was found to be a reason for stopping breastfeeding (Claesson et al. 2018).

### 3.3 What types and content of support offered by health care professionals, peer supporters, and family members during and after pregnancy could increase breastfeeding initiation and continuation among women who are overweight or obese?

#### 3.3.1 Support from health care professionals

Jarlenski et al. (2014) found an association between health care professionals' support/favour for/of exclusive breastfeeding and overall breastfeeding initiation and duration. In the overall sample, after adjusting for covariates, health care professionals' support/favour (defined as “physicians” and “nonphysicians”) for/of exclusive breastfeeding was associated with an 8.5% increased probability of breastfeeding initiation (95% CI [6.3, 10.7], *p* < .01, and a 13.2% increase in probability of continuing breastfeeding to 6 months or longer (95% CI [9.1, 17.3], *p* < .01), independent of whether women were with or without obesity.

#### 3.3.2 Support from partners, family members, and friends

The influence of partners, family members, and friends on breastfeeding outcomes was explored by Mok et al. (2008) and Keely et al. (2015). Mok et al. (2008) reported that a woman's choice of how to feed her infant was influenced by feeding practices of close family members, as well as her partner's opinion. Keely et al. (2015) confirmed that close family was an important source of practical support and influence on decisions to continue breastfeeding, especially if a relative had previously successfully breastfed. Conversely, a woman's partner could influence a woman's decision to introduce formula milk, often in response to breastfeeding problems: “He kept saying, ‘Just . . . if it's that sore . . . just stop, because it's not the end of the world'. He was like, ‘There's no point torturing yourself for it'” (p. 536).

### 3.4 What are health care professionals', peer supporters', and family members' perceptions of providing breastfeeding support and how do they perceive their role in this?

Only one paper presented perspectives of relevant health care professionals (Garner et al., 2014). Some described multiple challenges, with women's care described as “hugely time‐consuming” (p. 506) due to obesity‐related comorbidities, women's more limited mobility, increased physical effort, and need for more frequent breastfeeding assistance: “We dread those patients” because “it's so hard to take care of them” (p. 507). They perceived women's lack of confidence as major psychosocial barriers to breastfeeding and large breasts as a major physical challenge. Health care professionals described awareness of obesity stigma and efforts to be sensitive including “using gentle language and asking permission to touch” (p. 507). Nevertheless, it was clear that obesity caused embarrassment in the patient/health care professional relationship, with implicit stigma in the way professionals communicated with women with obesity or responded to their questions (Garner et al., 2014). They claimed to treat all women the same way but breastfeeding discussions with women with obesity were frequently not a priority. They considered that more education on how to support women with obesity to breastfeed was required, and highlighted care could be improved by better preparing women for breastfeeding during pregnancy, including positions for breastfeeding. Possible benefits of providing postnatal home contacts were also mentioned. No studies were identified which had specifically described peer supporters, family members, or partners' perceptions.

## 4 DISCUSSION

This review examined both qualitative and quantitative evidence of breastfeeding practices and breastfeeding support experienced by women who are overweight or obese, their perceptions of support they received, and what type of support impacted on breastfeeding initiation and duration. The perceptions of those who supported women to breastfeed were also considered. Sixteen papers were included, all from high‐income countries. Only two studies (Garner et al., 2014; McKenzie et al., 2018) achieved a full quality assessment (i.e., CASP) score. Findings highlighted that breastfeeding support for women with higher BMIs is a complex, multifactorial issue which, if women's needs are to be met, has to take account of physical, physiological and psychological challenges, and system factors including postnatal ward environment and clinical education.

### 4.1 Physical and physiological challenges

The findings of this current review echo many of the physical and physiological challenges identified by Babendure et al. (2015). Babendure et al. (2015) investigated factors that reduced breastfeeding incidence, duration, and exclusivity and evaluated interventions to increase breastfeeding among women with obesity (BMI ≥ 30 kg/m^2^). Our review, which also included studies of women who were overweight (BMI ≥ 25 kg/m^2^), provides additional qualitative evidence to better place findings into context of the type of support and environment of care which could benefit women with higher BMIs. Physical challenges such as women having larger breasts and difficulties with attaching their babies to the breast impacted on their breastfeeding success (Claesson et al., 2018; Garner et al., 2017; Jarlenski et al., 2014; Massov, 2015). This, combined with lack of practical support from health care providers (Garner et al., 2014; Kair & Colaizy, 2016b; Claesson et al., 2018), highlights an important gap in how women are informed about positions to commence feeding.

As chances of spontaneous vaginal birth diminish with increasing BMI (Leddy, Power, & Schulkin, 2008; Nilses, Persson, Lindkvist, Petersson, & Mogren, 2017), clinician training to provide tailored breastfeeding support in hospital and at home should be a priority for all maternity care providers. Poor support generally for breastfeeding following caesarean birth was highlighted in a recent systematic review (Beake, Bick, Narracott, & Chang, 2017). The current review contributes further evidence that women with higher BMIs who have caesarean births not only have problems with mechanical aspects of breastfeeding but also have consequences of post‐operative recovery in hospital environments where clinicians may be unable, or unwilling, to offer the support and advice they need, or protect their privacy. Tailored support could also prevent women from developing sore, cracked nipples, which were more common among women with higher BMIs (e.g., Kair & Colaizy, 2016a).

Several studies (Mok et al., 2008; Jarlenski et al., 2014; Kair & Colaizy, 2016a; Massov, 2015; O'Sullivan et al., 2015; Claesson et al., 2018) reported that women who were overweight or obese were more likely to report insufficient breastmilk as a reason for early cessation of breastfeeding than women with normal BMIs. This is one of the most commonly reported reasons for early cessation generally in high‐income country settings, including Australia, UK, and Canada (Brown, Dodds, Legge, Bryanton, & Semenic, 2014; McAndrew et al., 2012; Newby & Davies, 2016) and may be an indicator of other reasons for stopping breastfeeding, as insufficient milk of itself is unlikely if women are breastfeeding effectively. However, the included studies did not fully explore this reason or define what “insufficient breastmilk” actually meant. Reasons for stopping breastfeeding are likely to be complex, and “insufficient milk” may seem to be a more socially acceptable reason that women feel able to report. The issue of insufficient milk warrants further investigation among all breastfeeding women but particularly for women with higher BMIs. It is not known to what extent perceived lack of breast milk in these women reflects physiological reasons (e.g., differences in adipose tissue), compounded by poor infant sucking due to mechanical barriers, such as poor latching and positioning on large breasts, and/or a consequence of inadequate postnatal support and information.

There is evidence that women in some cases do experience delayed onset of lactation (DoL). DoL was explored in one included paper which found BMI, larger infant birthweight, and older maternal age were associated with DoL (Nommsen‐Rivers et al., 2010). Obesity as a predictor of delayed lactogenesis II (the onset of copious milk production) was found in a later study by Preusting, Brumley, Odibo, Spatz, and Louise (2017), and although not a focus of this review, further research into better understanding reasons for DoL are urgently needed.

Medical complications such as caesarean birth or prolonged labour could inhibit oxytocin, a crucial hormone triggering lactation onset, with a potential link between lactogenesis and decreased insulin production. Further investigation into physiological differences which may exist because of higher BMIs and/or mode of birth is needed. In the interim, tailored, timely, and individualised breastfeeding support, including advice on expressing/pumping breastmilk, should be offered to women with higher BMIs to prevent potential DoL particularly following a caesarean birth.

### 4.2 Psychosocial challenges

As in Lyons, Currie, Peters, Lavender, and Smith's (2018) review of the association between psychological factors and breastfeeding behaviour, psychosocial barriers to breastfeeding were also identified from women's perspectives, most notably women's perceived poor body image (Hauff & Demerath, 2012; Keely et al., 2015; Massov, 2015; Mok et al., 2008; Swanson et al., 2017). Body image appears to be an important factor when considering challenges to increase breastfeeding initiation and duration among women with higher BMIs (Hauff & Demerath, 2012; Swanson et al., 2017). Embarrassment at breastfeeding in public influenced some women to choose formula feeding (Massov, 2015; Keely et al., 2015; Hauff & Demerath, 2012; Claesson et al., 2018; McKenzie et al., 2018). In Western societies, where there is a media obsession with post‐birth bodies of celebrities, women who are overweight or obese may be even less keen to expose parts of their body to breastfeed in front of others (Hauff & Demerath, 2012) due to stigma about their body image. As images of women breastfeeding are unlikely to include women with higher BMIs, “normalising” breastfeeding among these groups may be difficult to achieve. This highlights that clinicians need to prioritise timing and content of support offered which addresses stigma or embarrassment they or the woman may feel.

It is possible that women who could benefit from tailored support for breastfeeding are reluctant to seek help because of concerns about the stigma of their weight: a similar situation to perinatal mental health where women have described being reluctant to report mental health problems because of being perceived as “bad mothers” (Moore, Ayers, & Drey, 2016). Attention needs to be given to the education of maternity care professionals, including strategies on how to avoid stigmatising women, development of effective communication skills, and evidence of why breastfeeding is so important for maternal and infant health. Research into how education on obesity can be best provided and supported by those on pre and post‐registration clinical training programmes in higher education institutions is needed (Olander & Scammell, 2015).

Women's attitudes and confidence in their ability to breastfeed were also important (Hauff et al., 2014). Intervention studies aiming to improve breastfeeding rates among women with obesity by increasing breastfeeding self‐efficacy (aka confidence) were unsuccessful (Chapman et al., 2013), and it is clear that interventions that address the multi‐faceted challenges of breastfeeding as identified in this review are needed.

### 4.3 Impact and success of support offered

Another aim of the current review was to consider the impact and success of support offered to women who were overweight or obese by health care professionals, peer supporters, and family members. The beneficial effect of positive support was described (Claesson et al., 2018; Keely et al., 2015) as was the effect of negative support (Claesson et al., 2018; Massov, 2015). The findings highlight that support has to be tailored to women's individual needs. Women with higher BMIs were less likely to seek support despite experiencing greater breastfeeding problems (Mok et al., 2008). If negative attitudes are encountered, the likelihood of seeking the health support they need is likely to reduce further. In terms of practical support, advice that larger beds and chairs be used postnatally to help women who are overweight or obese find a comfortable, successful breastfeeding position could be considered (Jevitt, Hernandez, & Groër, 2007), as could use of breastfeeding support plans tailored to individual women's needs.

Women's partners may reaffirm perceptions of insufficient breast milk supply through a desire to support a woman who is anxious or upset and actively encourage her to stop breastfeeding (Keely et al., 2015). Partner support is crucial to women's decisions about infant feeding (Littman, VanderBrug Medendorp, & Goldfarb, 1994), and involvement of partners in antenatal discussions on infant feeding could reduce well‐intentioned but negative influences. No research was identified for inclusion in this review which addressed partners' and family's views, an important evidence gap in terms of supporting women with higher BMIs.

Women with high BMIs received insufficient breastfeeding information and support (Claesson et al., 2018; Kair & Colaizy, 2016b; Keely et al., 2015; Massov, 2015). Too few interventions have been developed, implemented, and evaluated on support for breastfeeding among women with medically complex pregnancies, and no intervention studies published since 2014 were identified for inclusion in this review. A Cochrane review of interventions to support breastfeeding in healthy breastfeeding women and healthy term babies (which excluded women with overweight or obesity) found that when breastfeeding support was offered, duration and exclusivity of breastfeeding increased (McFadden et al., 2017).

## 5 STRENGTHS AND LIMITATIONS

The current review included experiences and perceptions of women with BMIs ≥ 25 kg/m^2^, those who supported them, and updated searches for relevant intervention studies published since 2014. However, no new intervention studies review met the review's inclusion and quality assessment criteria. We were also unable to identify or include any studies which had investigated family members' and breastfeeding peer supporters' experiences and perceptions. Only one study (Garner et al., 2014) which explored perspectives from health care professionals was included.

Most of the studies included had methodological limitations meaning some caution has to be applied to findings. Furthermore, findings may not be generalisable for several reasons. In majority of the included studies, women's BMIs were classified according to self‐reported weight and height which may not be as accurate as measured by study teams. Exclusion of non‐English language studies may have introduced selection bias. Nine papers were from the United States, a potential limitation given differences in populations, cultural attitudes to breastfeeding, settings, and context of care.

## 6 CONCLUSION

This review highlights the importance of planned, tailored support during and beyond pregnancy to enable women who are overweight or obese to commence and continue to breastfeed successfully and overcome barriers they encounter. Unless women with higher BMIs can access timely, tailored, and consistent support from maternity care professionals and their peers, uptake and duration of exclusive breastfeeding may continue to be lower to the continued detriment of maternal and infant health. That some health care professionals resented the extra support and time needed by women with higher BMIs needs to be urgently addressed by health care institutes and higher education institutions. The weakness of the evidence base highlights that further robust research, with large sample sizes, should be prioritised given the increasing burden of obesity among women of reproductive age worldwide.

## CONFLICTS OF INTEREST

The authors declare that they have no conflicts of interest.

## CONTRIBUTIONS

Y‐SC and DB initiated the concept for the review. AAG and Y‐SC conducted the search. AAG, Y‐SC, PD, SB, and DB assessed the selected text papers for eligibility and extracted data. Y‐SC and DB produced the initial draft of the manuscript and revised drafts following feedback from SB, PD, and AAG. SB aided in the revision of drafts. All authors read and approved the final version of the manuscript.
